# Supplementary figures and images for: Divisive attenuation based on noisy sensorimotor predictions accounts for excess variability in self-touch
Source: J Neurophysiol. Author manuscript; Available in PMC 2025 Jul 28. (PMC7617943; doi:10.1152/jn.00055.2025)

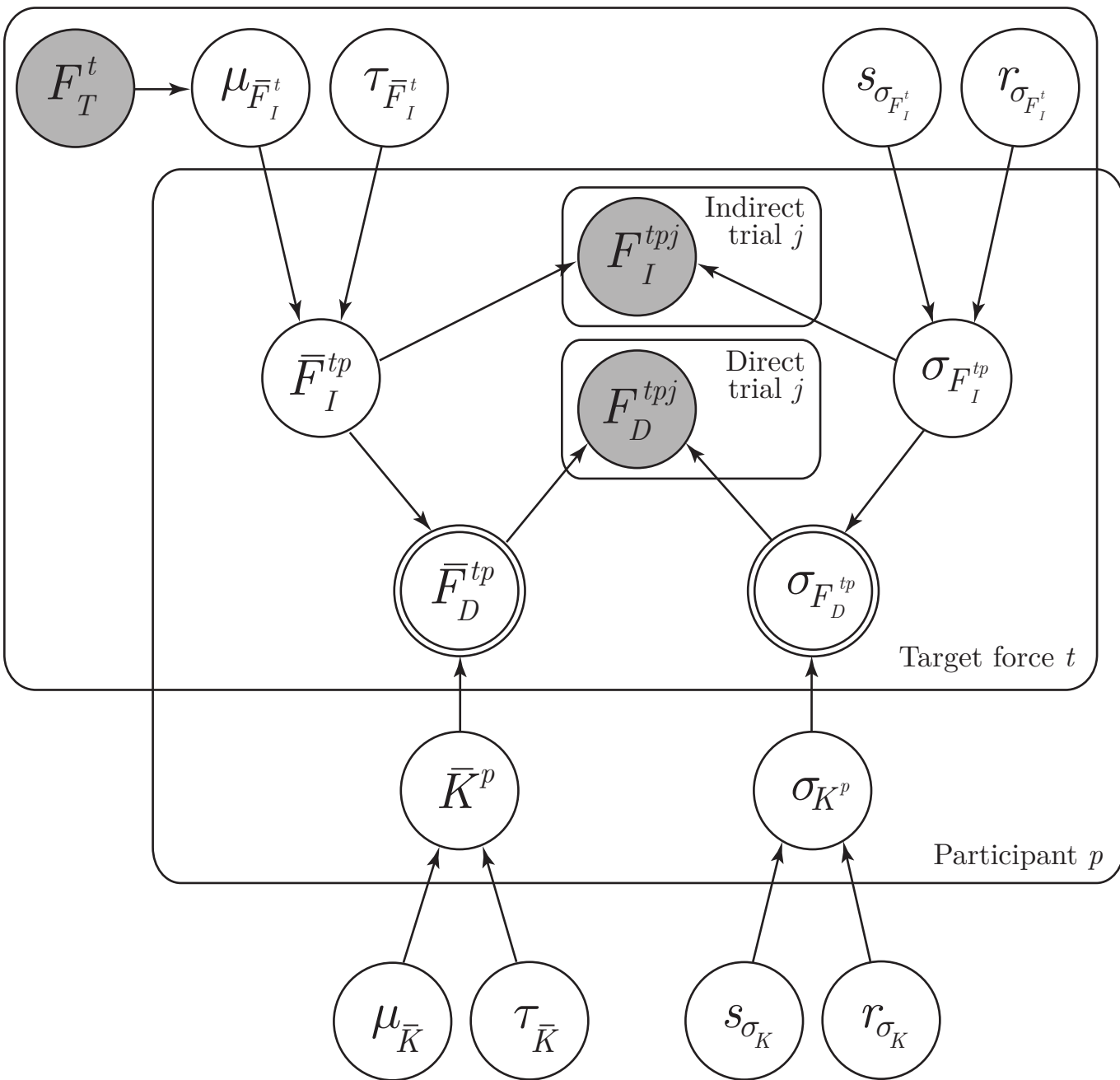

Supplement: Appendix Figure 1 [file EMS206575-supplement-Appendix_Figure_1.pdf]

Young

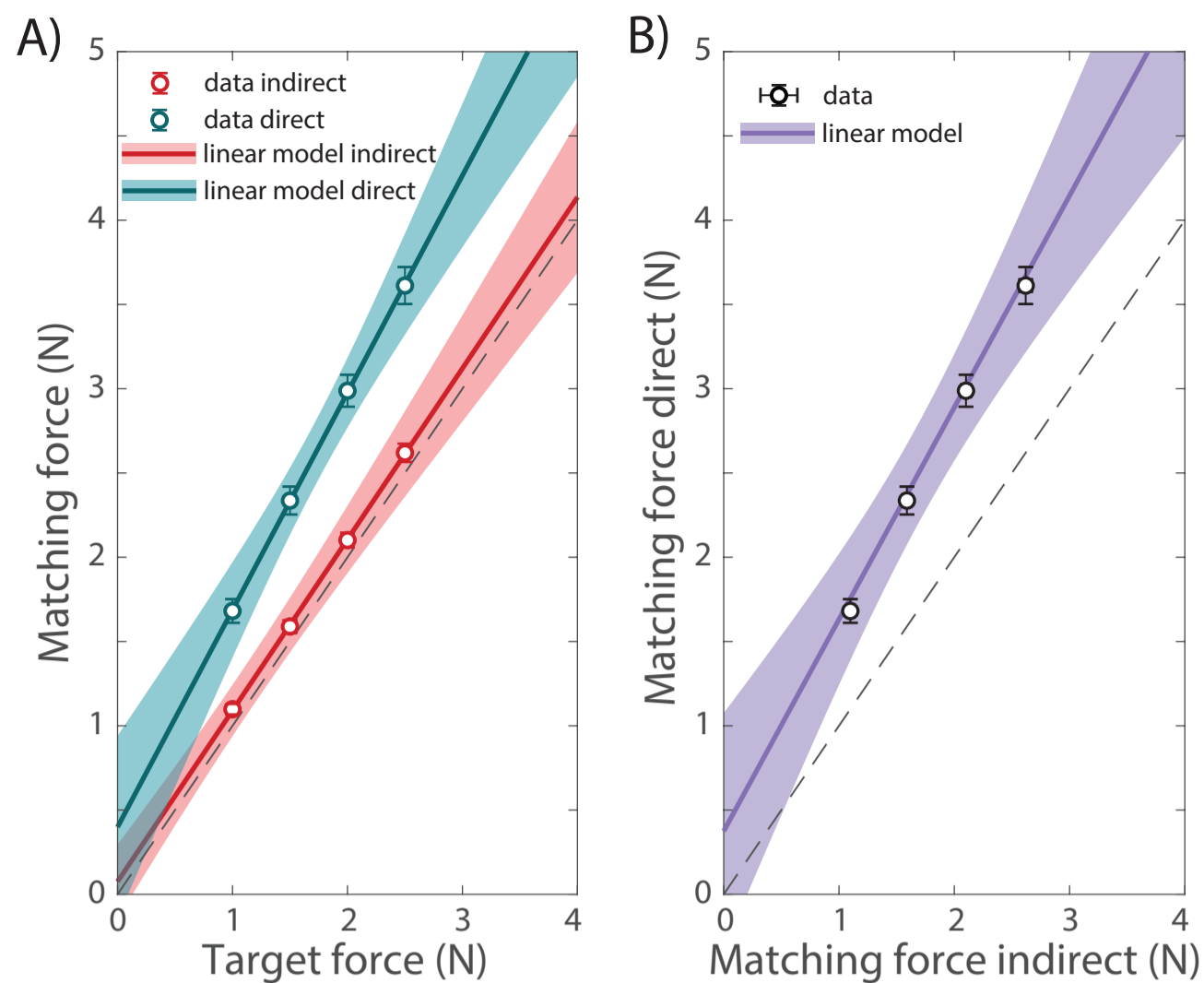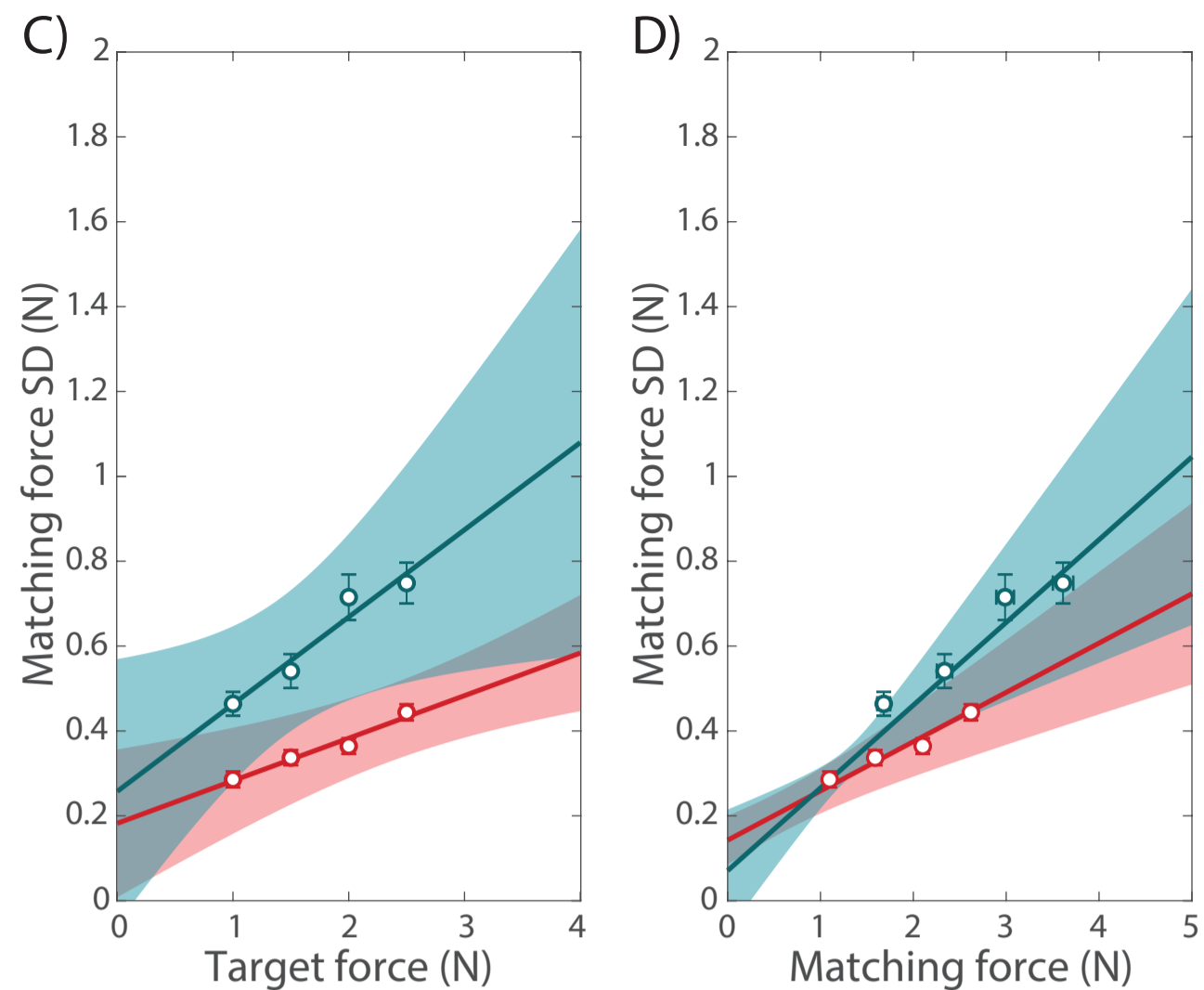

Middle

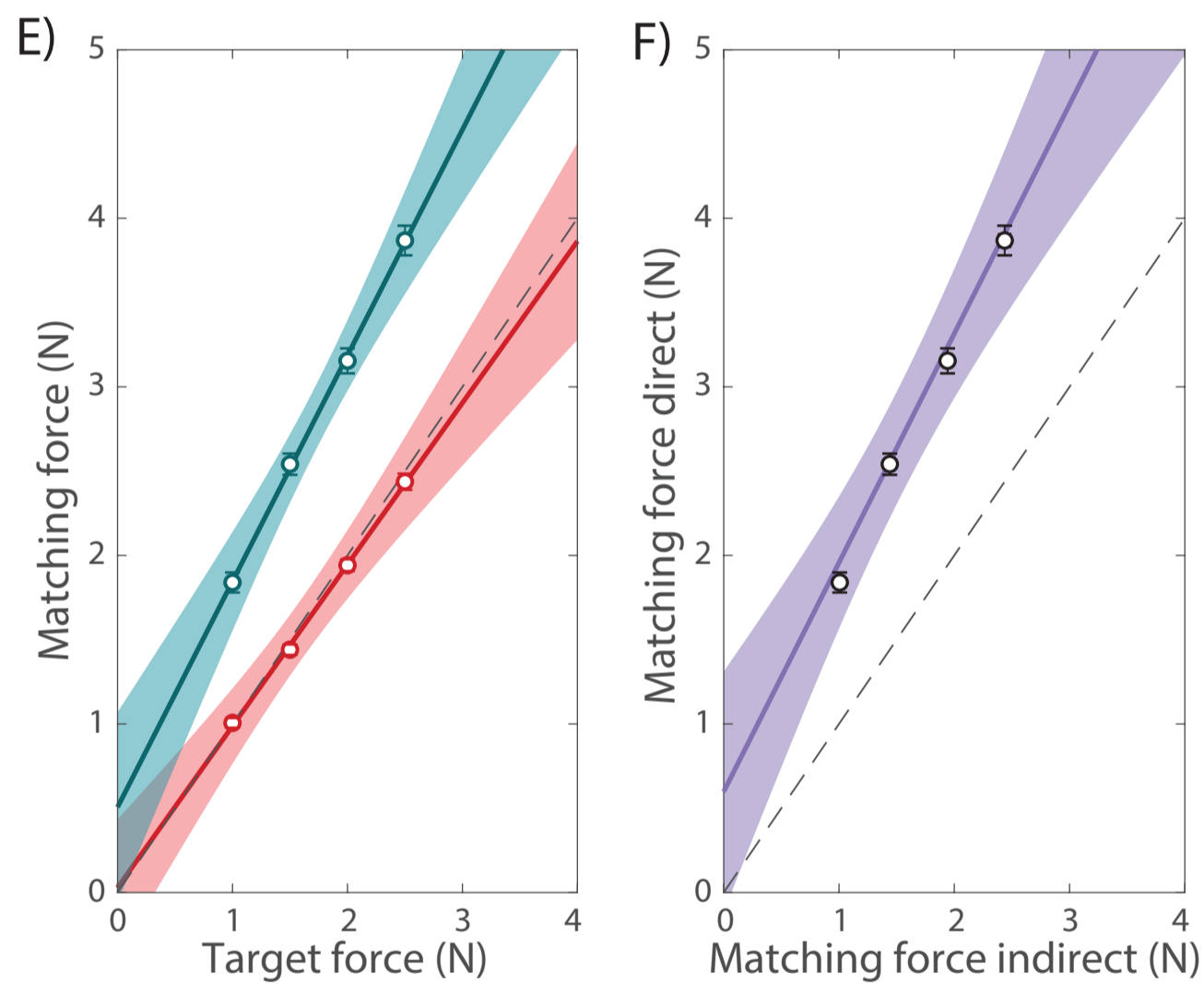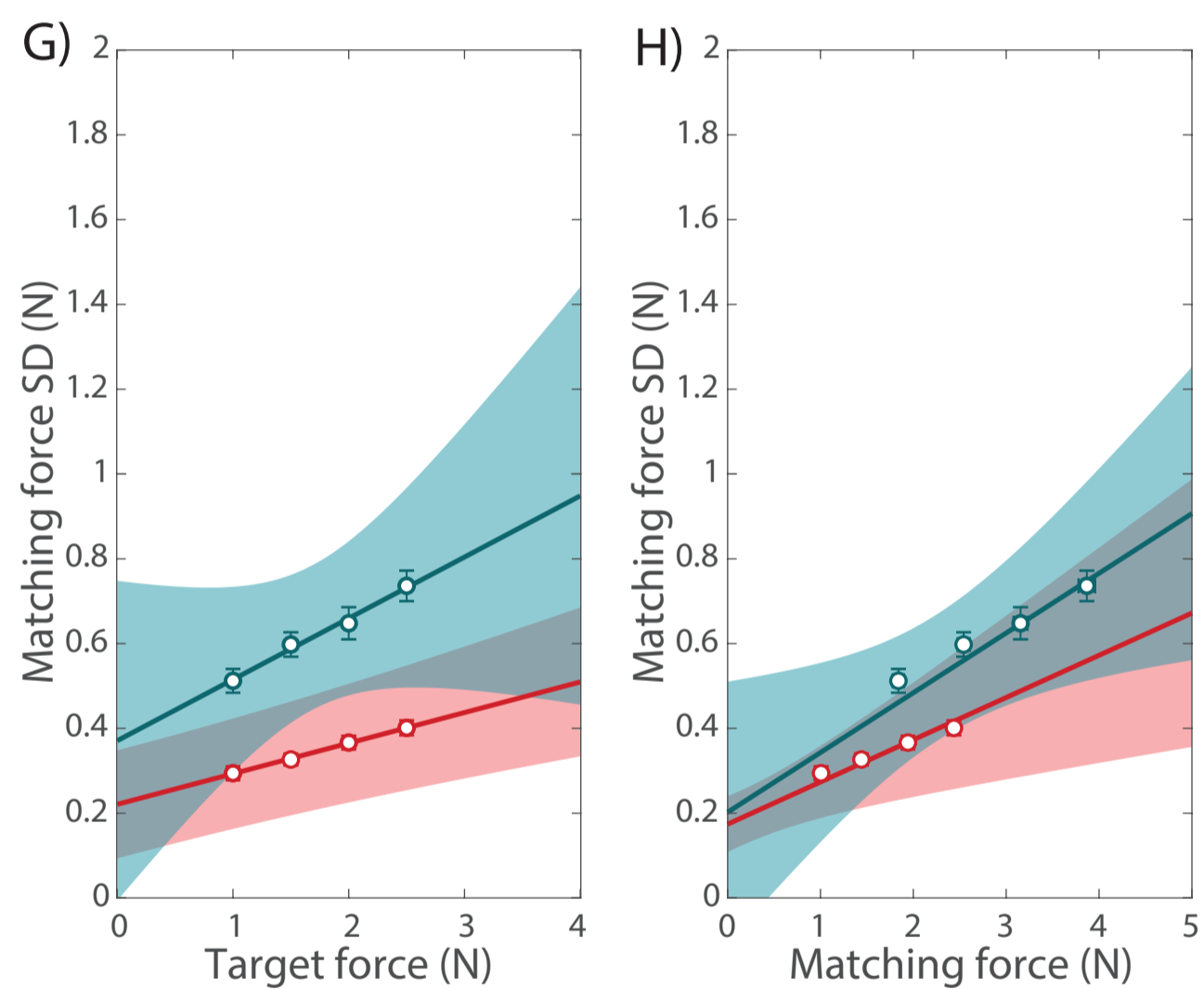

Older

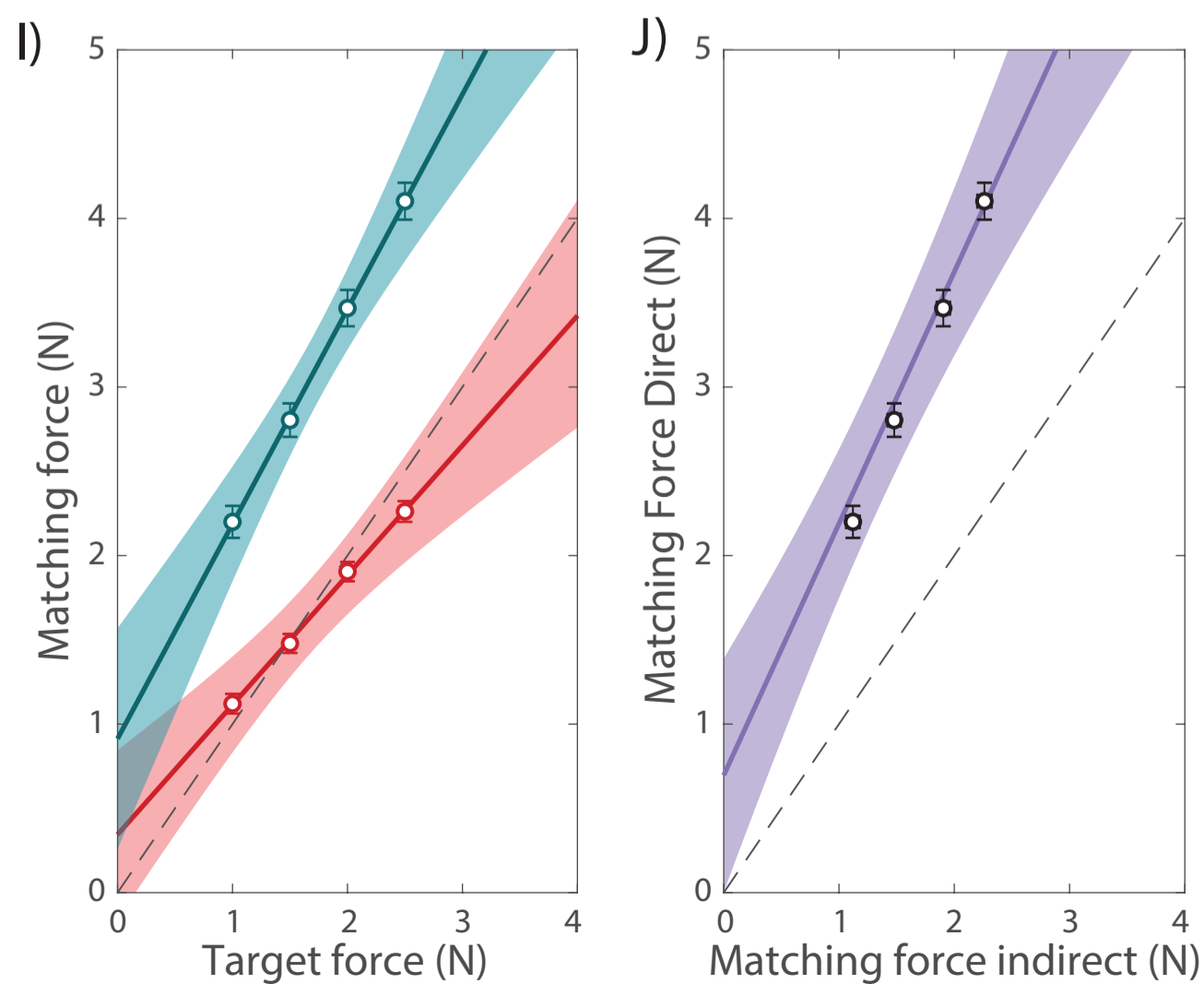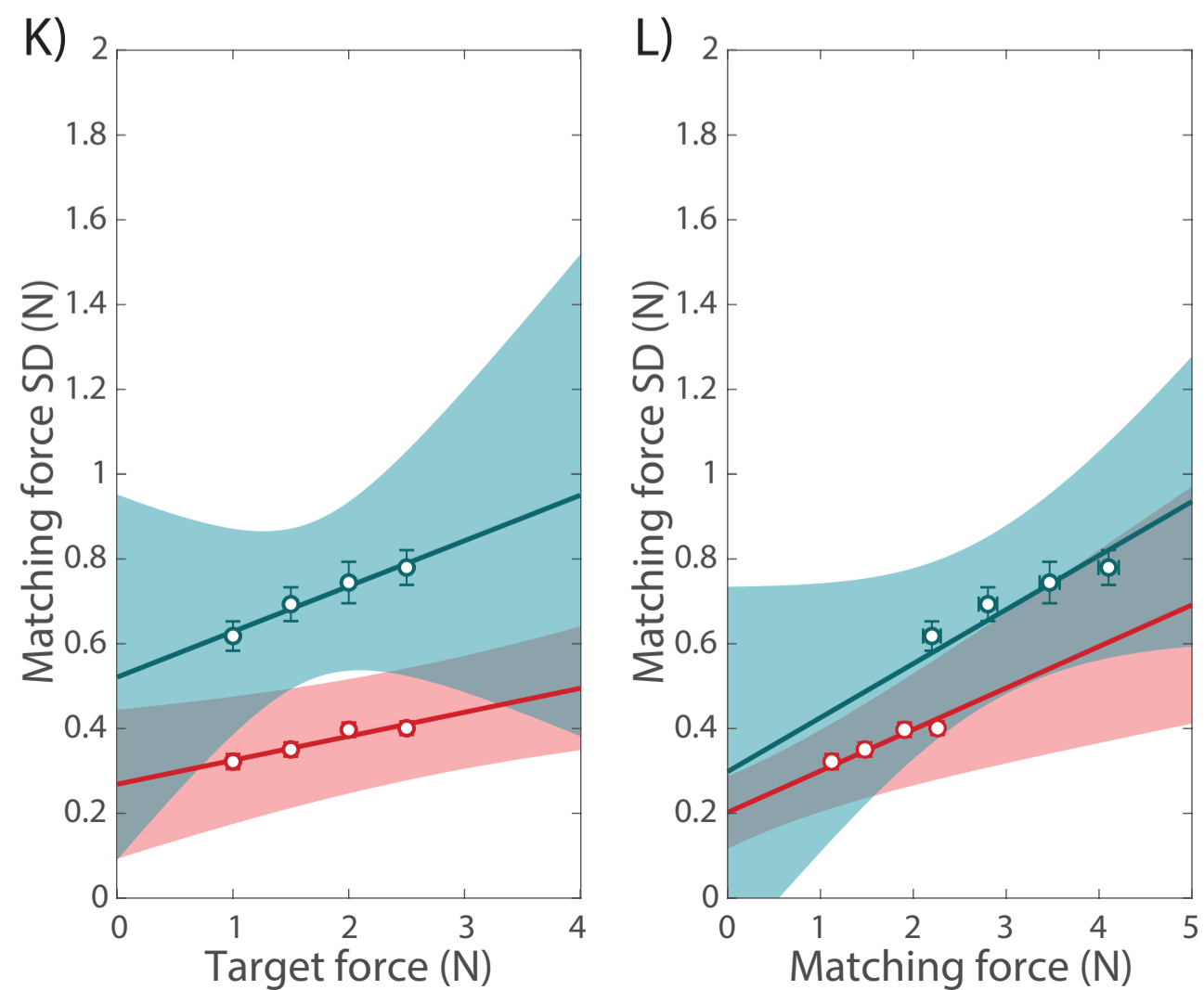

Supplement: Appendix Figure 2 [file EMS206575-supplement-Appendix_Figure_2.pdf]

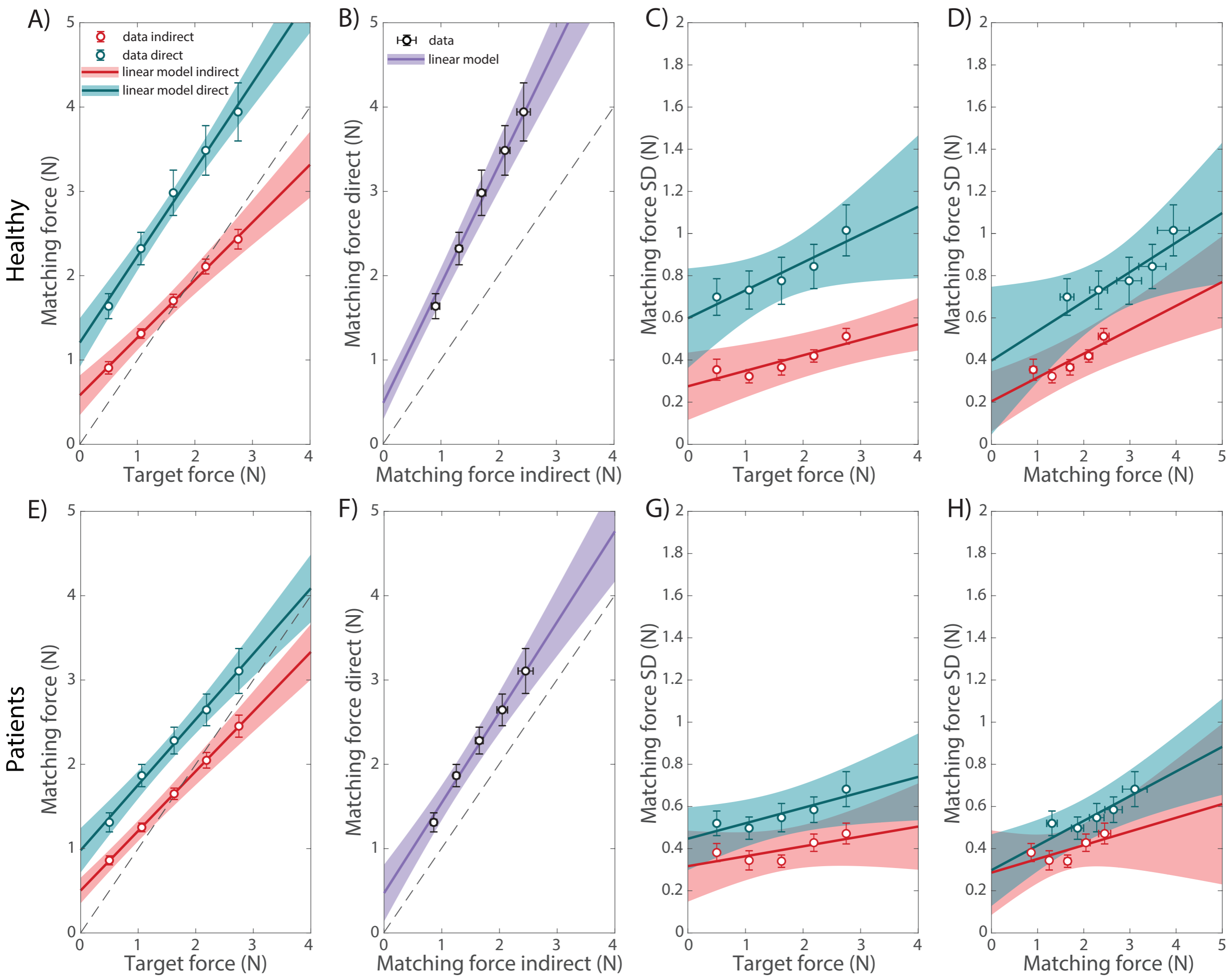

Supplement: Appendix Figure 3 [file EMS206575-supplement-Appendix_Figure_3.pdf]

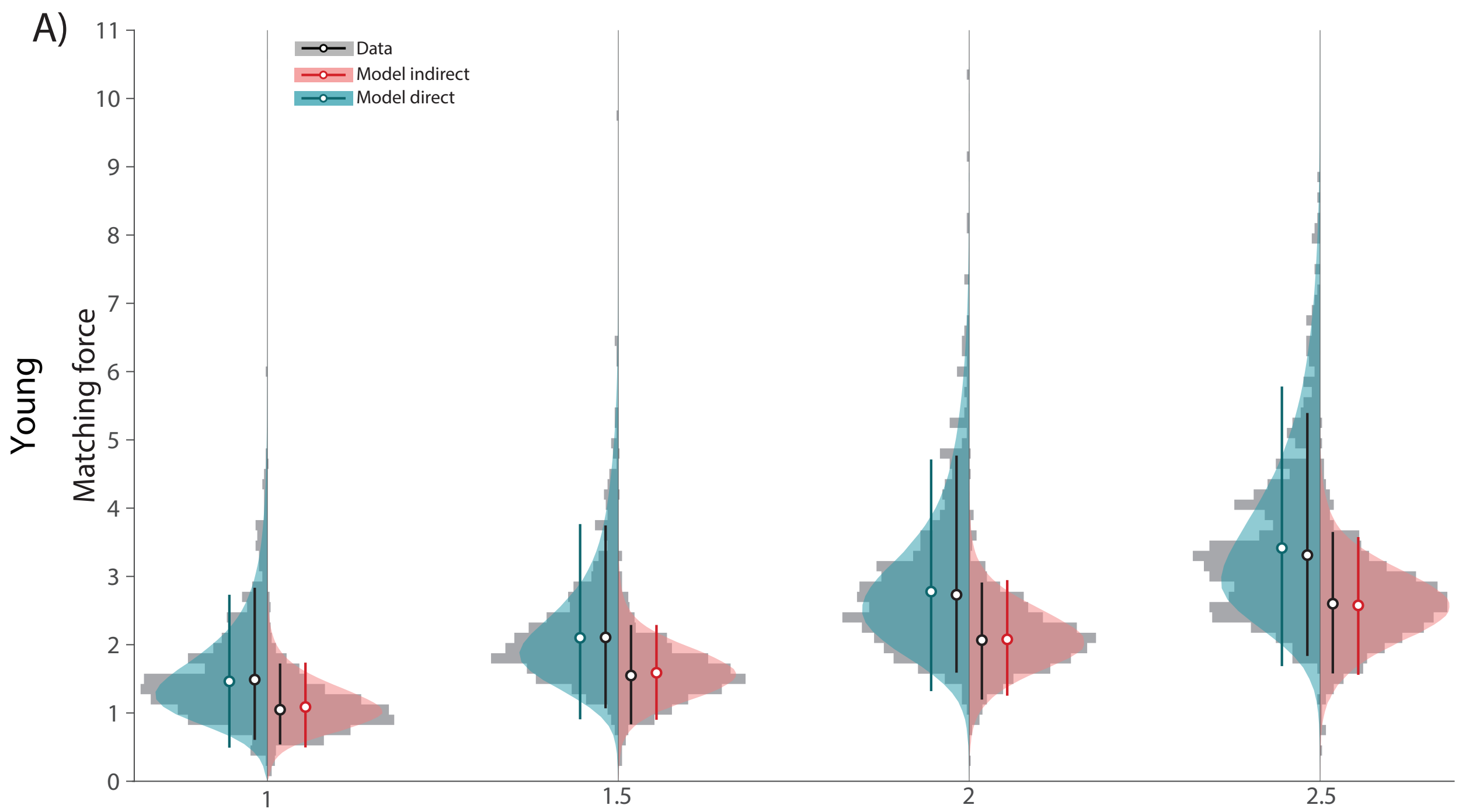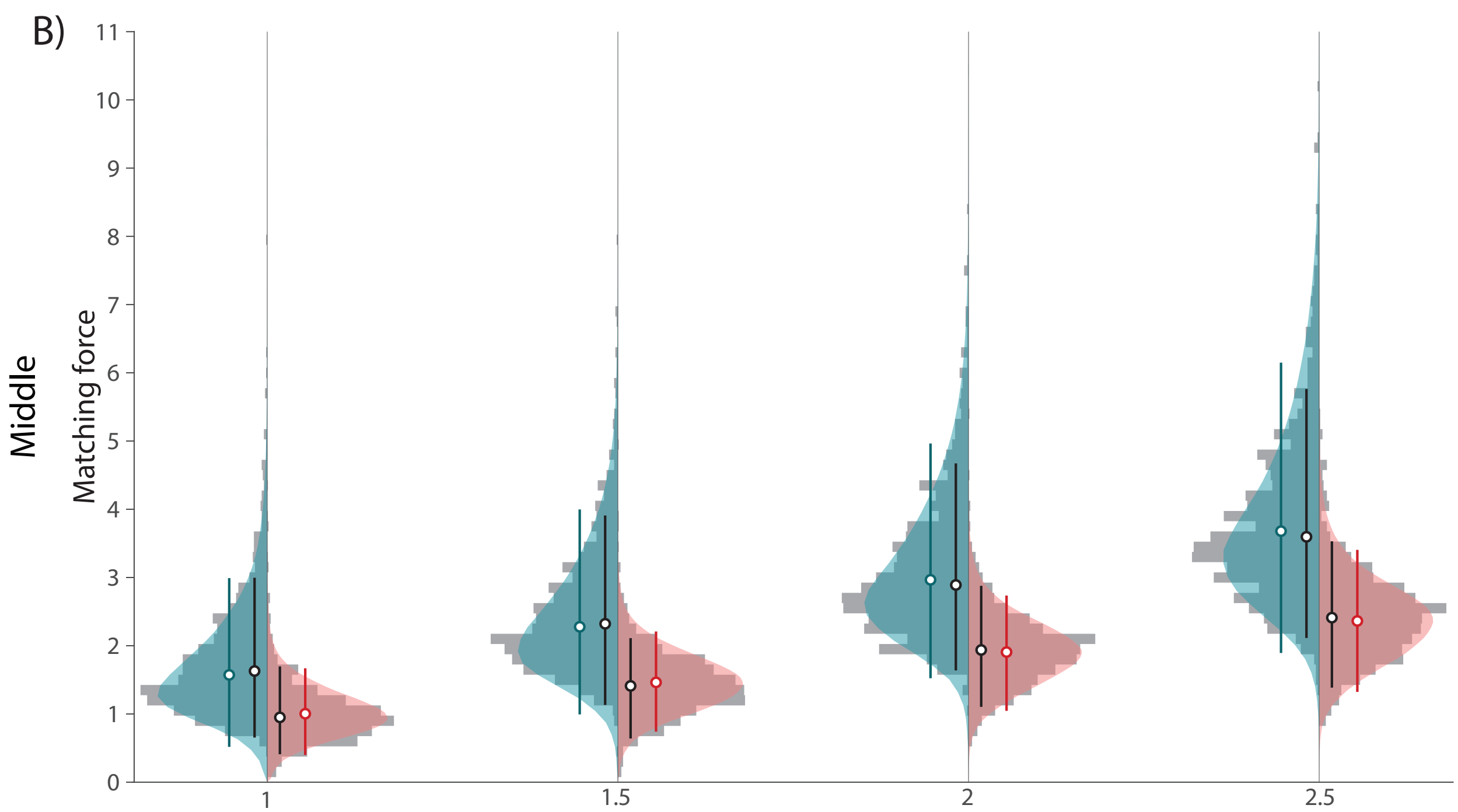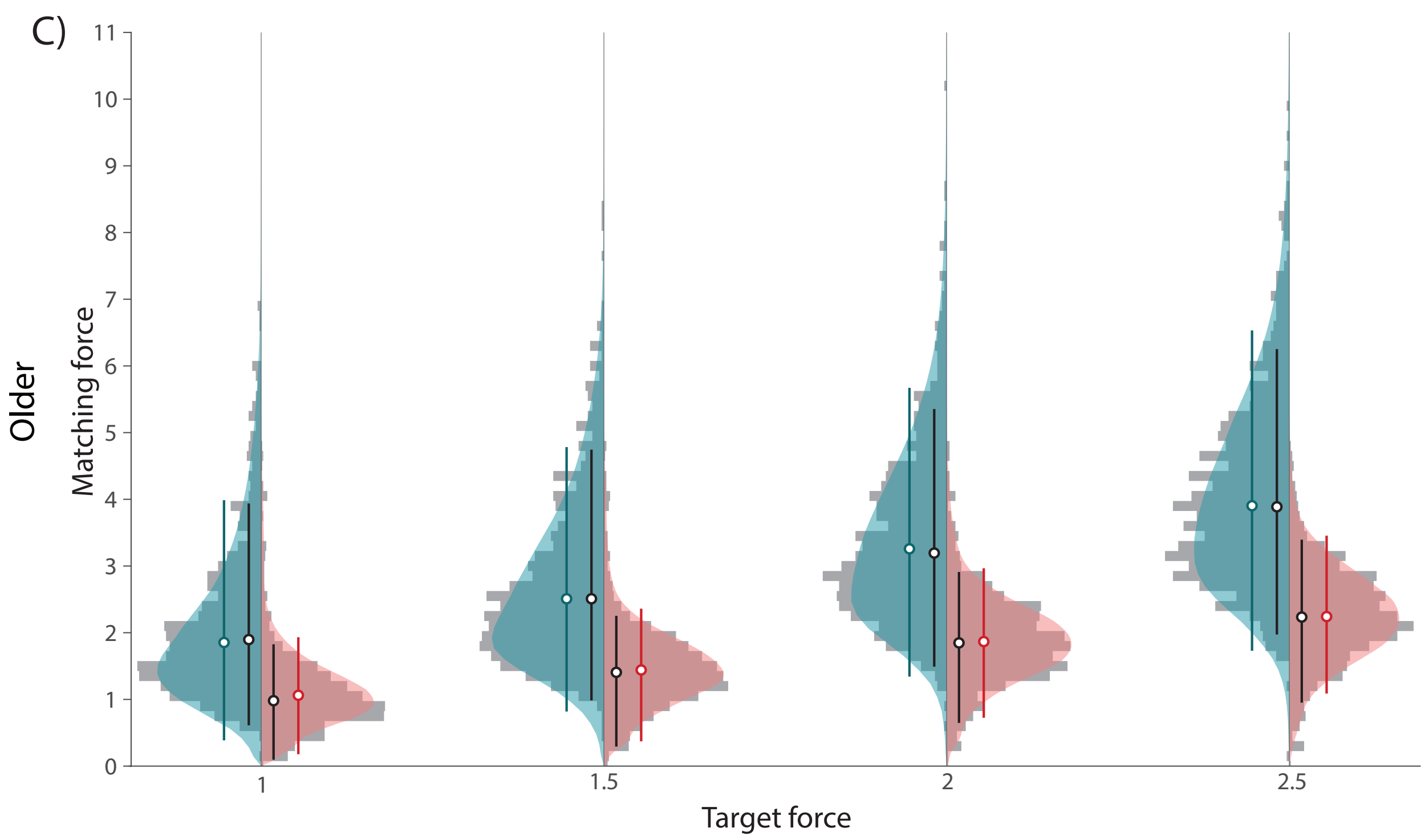

Supplement: Appendix Figure 4 [file EMS206575-supplement-Appendix_Figure_4.pdf]

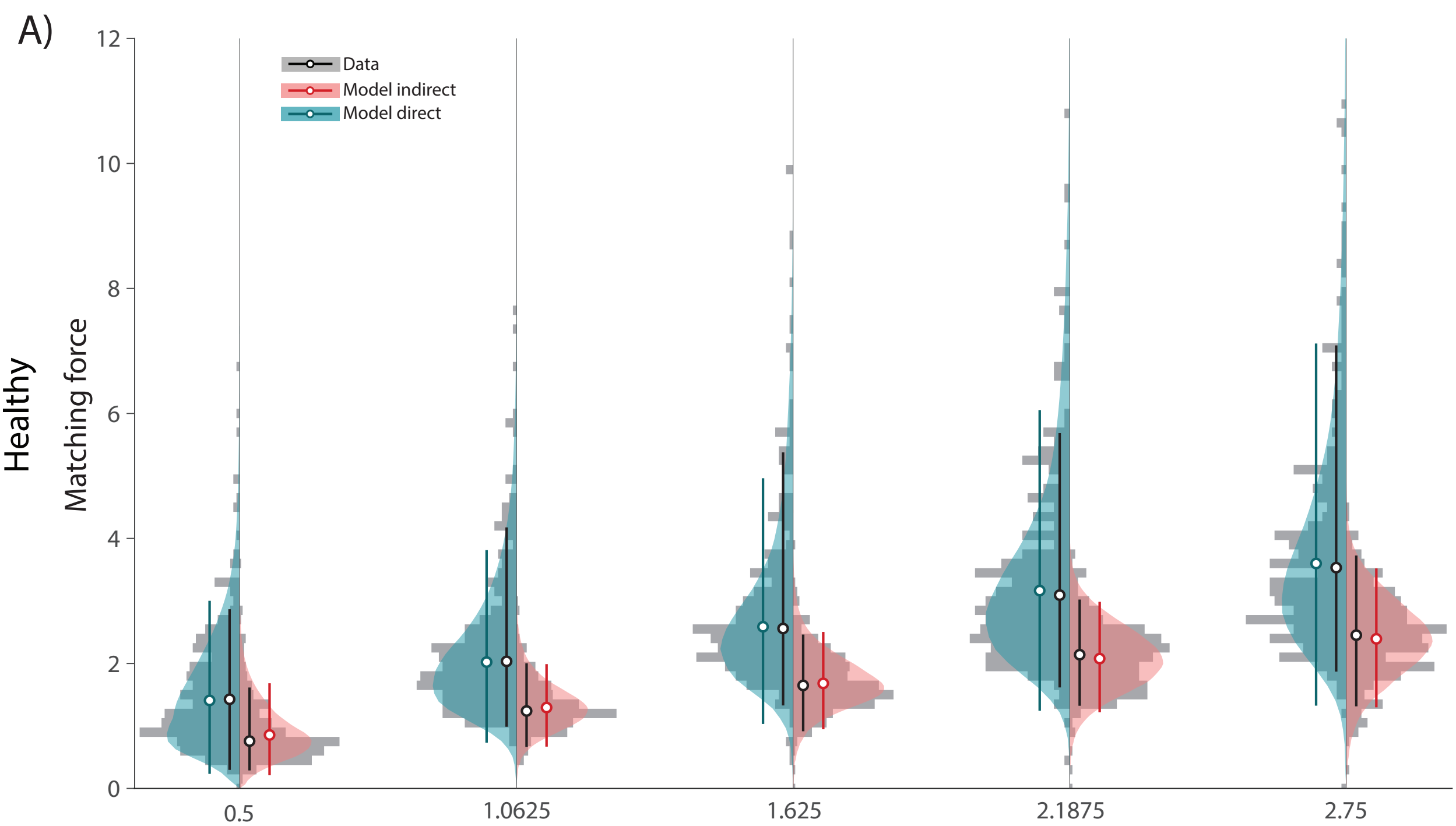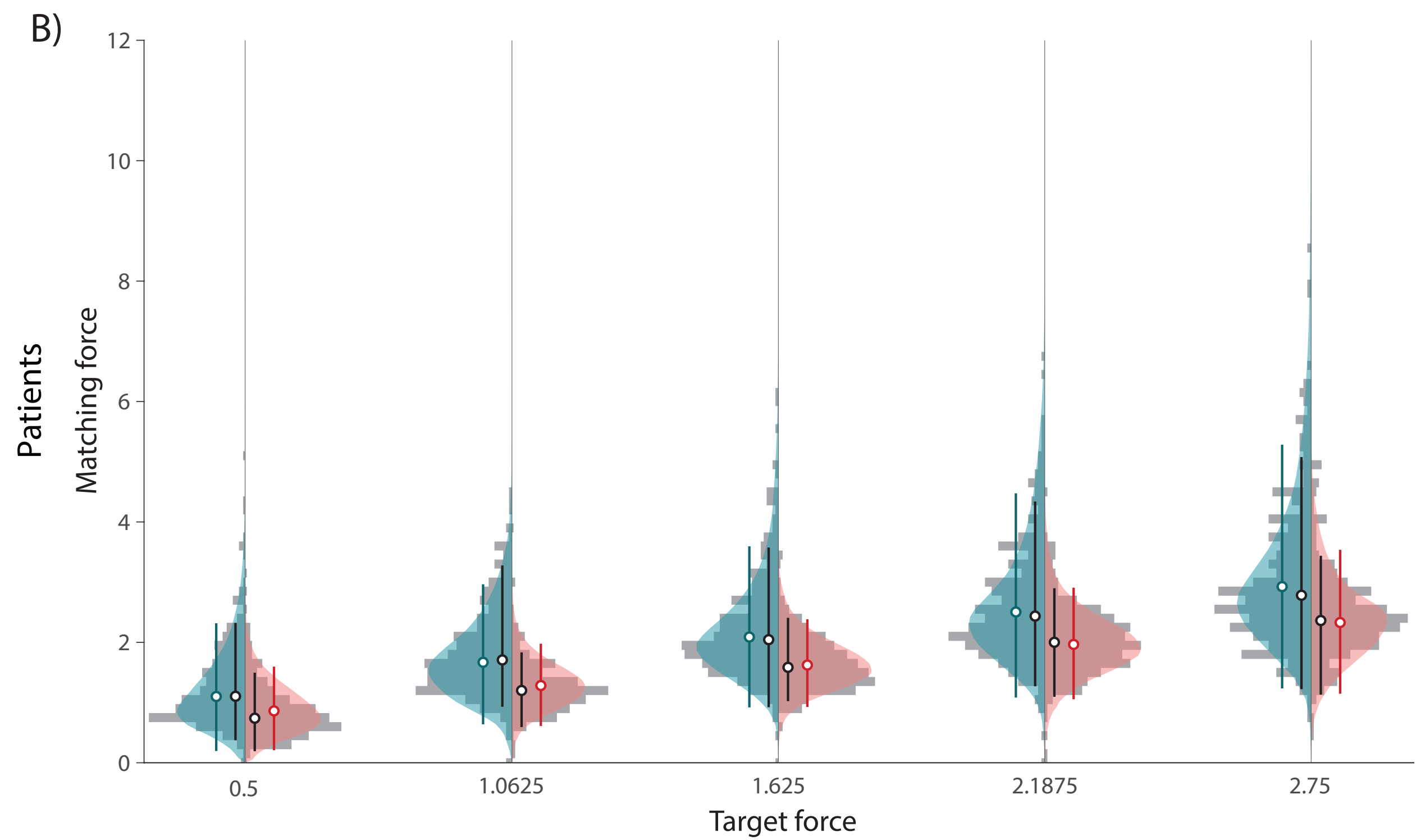

Supplement: Appendix Figure 5 [file EMS206575-supplement-Appendix_Figure_5.pdf]

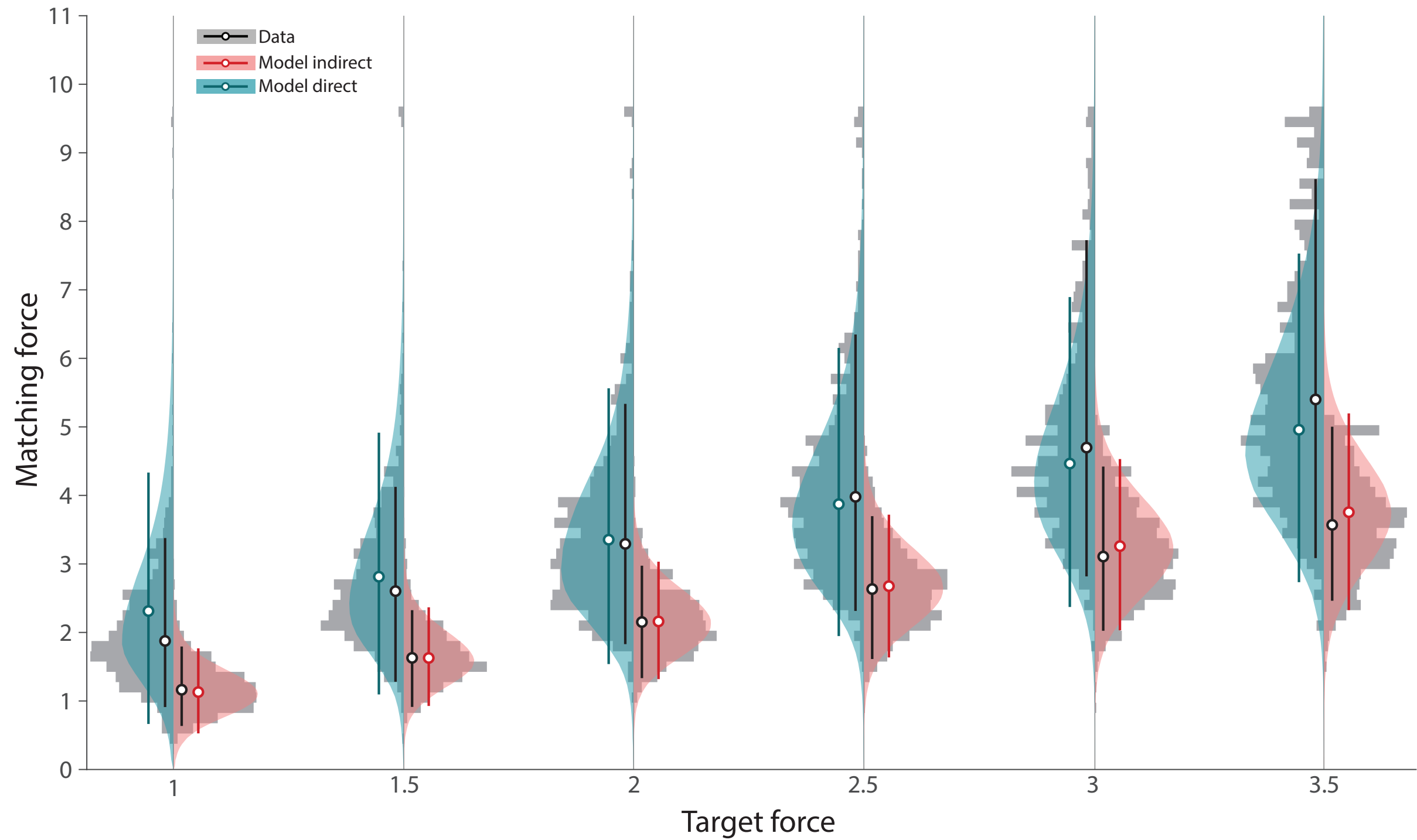

Supplement: Appendix Figure 6 [file EMS206575-supplement-Appendix_Figure_6.pdf]

1 Kilteni &amp; Ehrsson

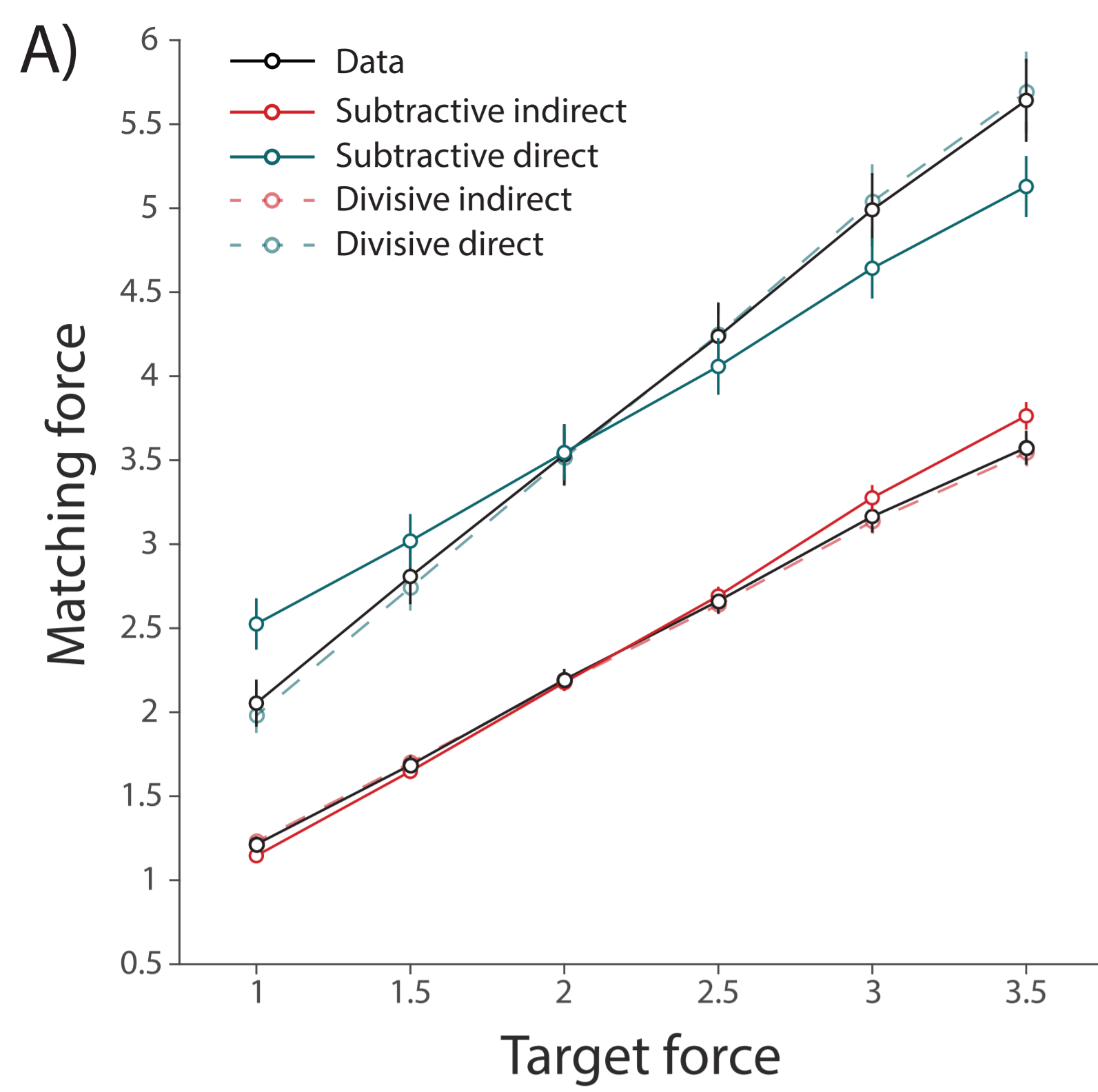

2 Wolpe et al. (2018)

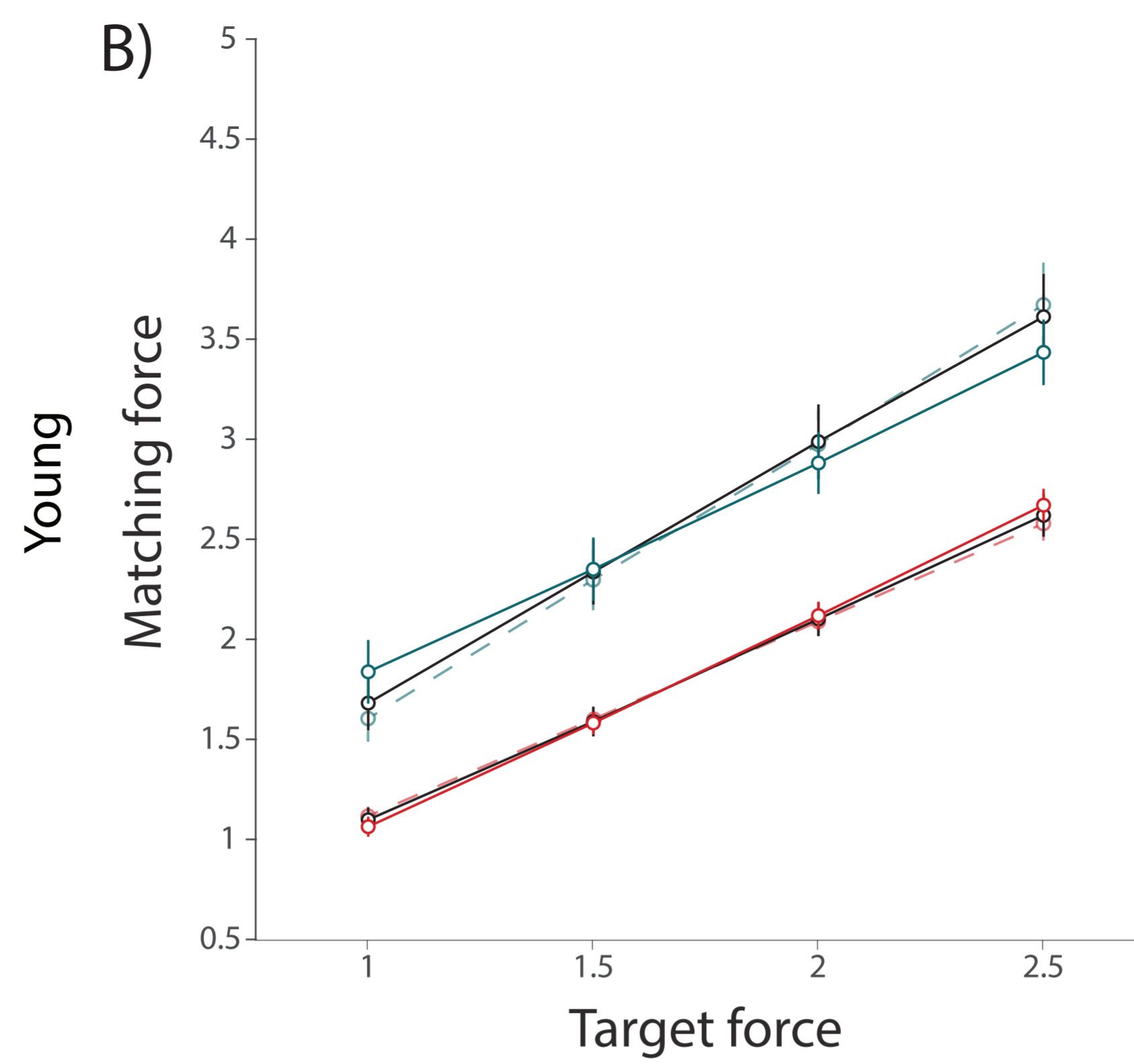

3 Shergill et al. (2015)

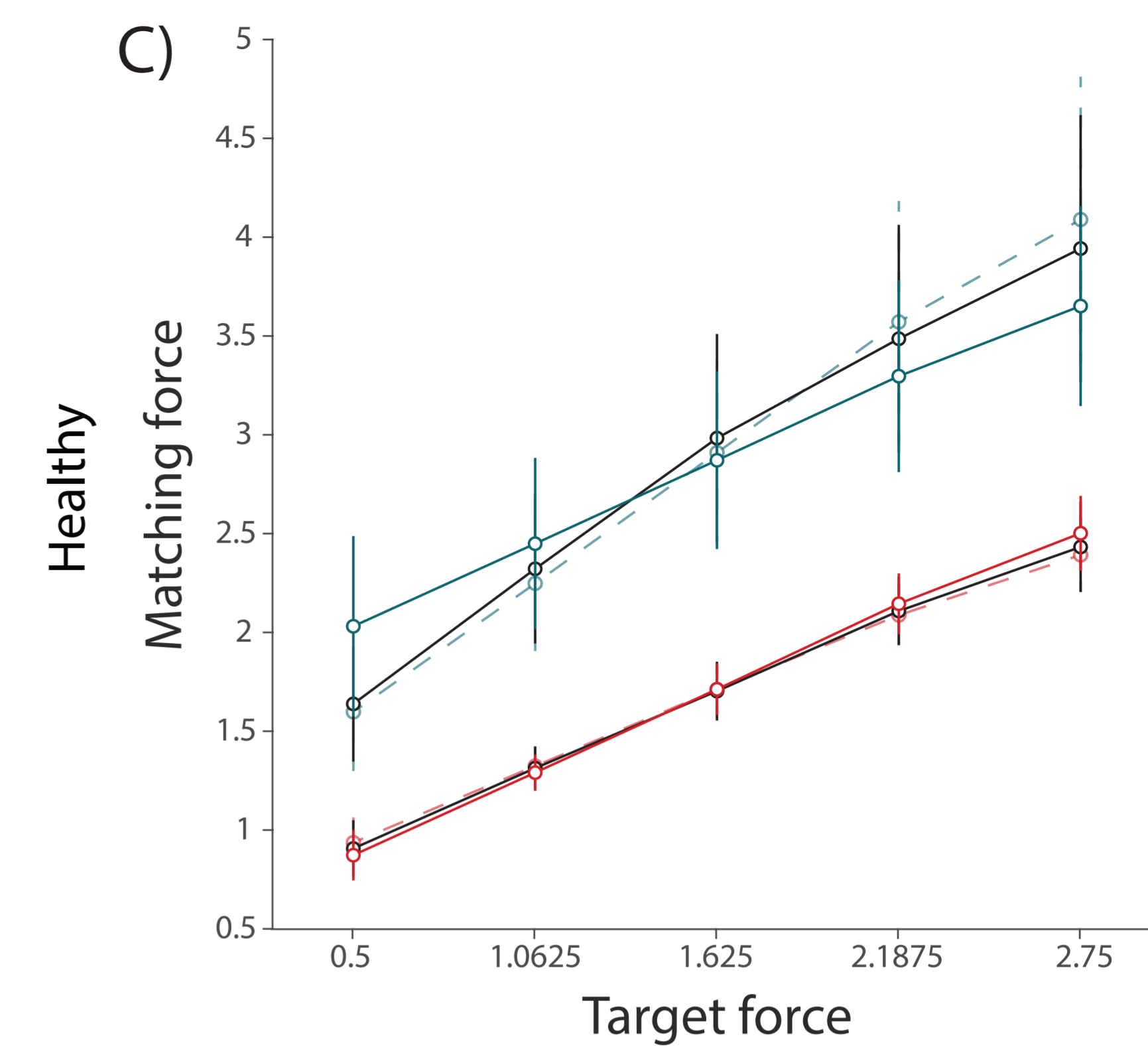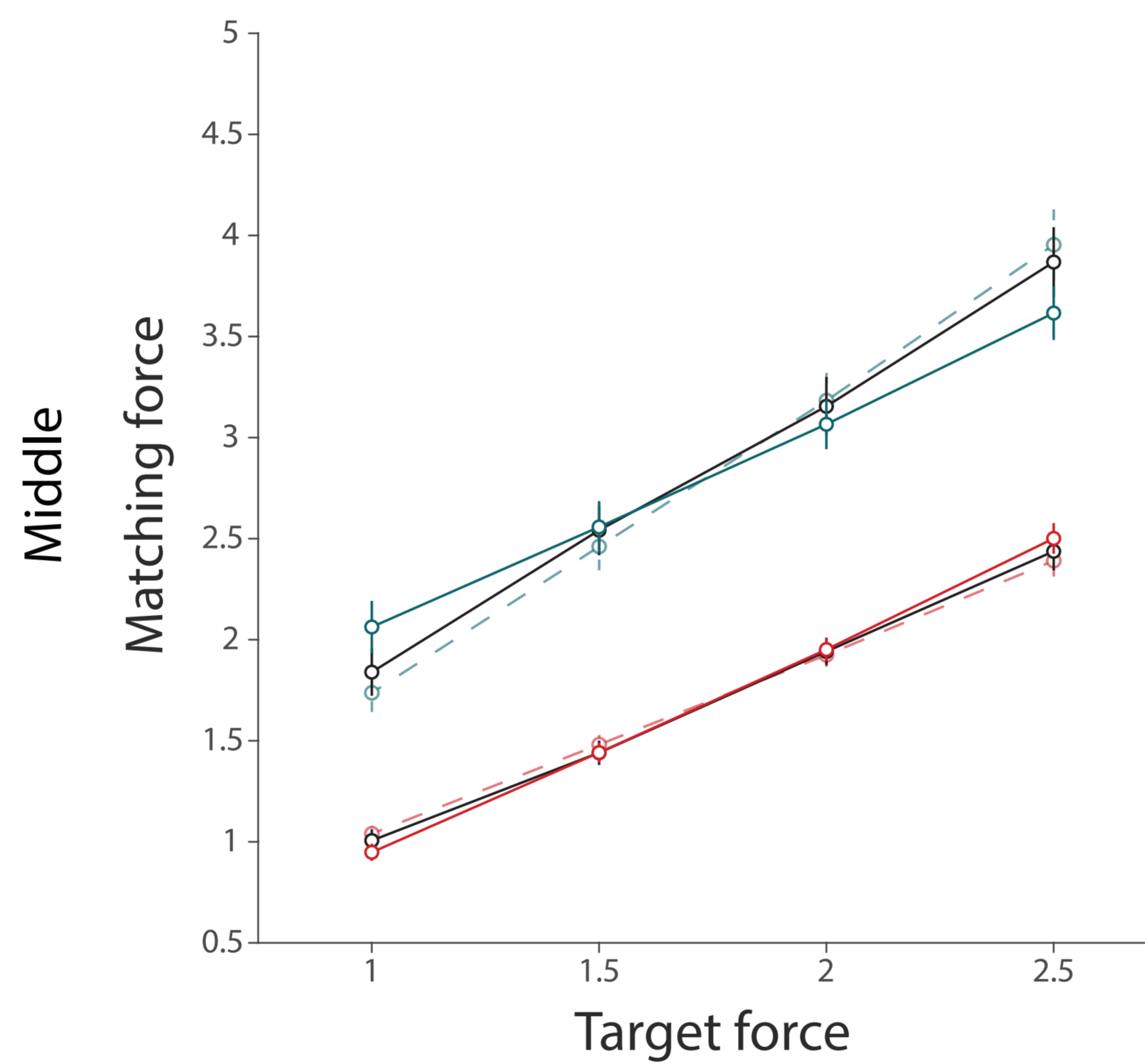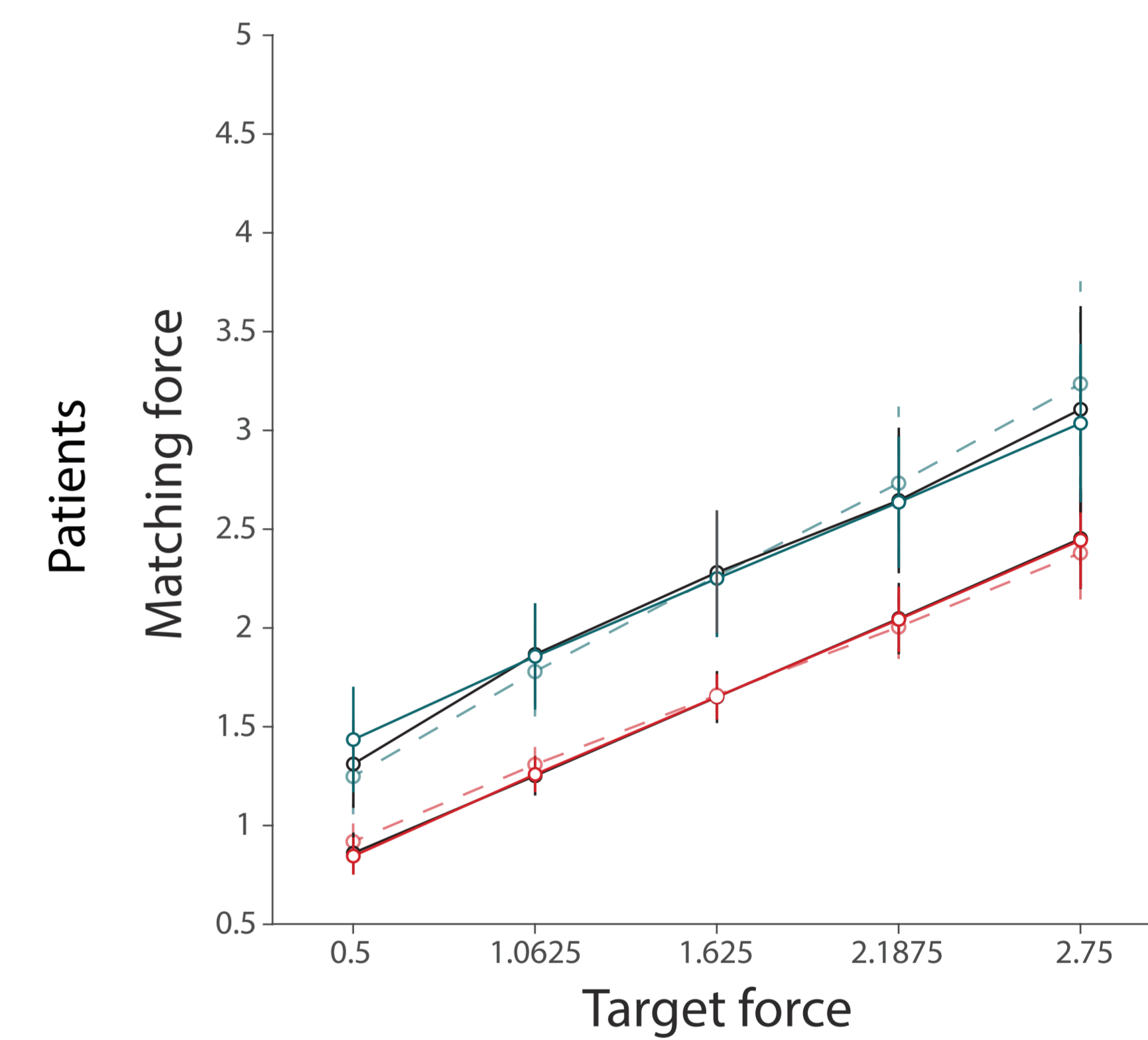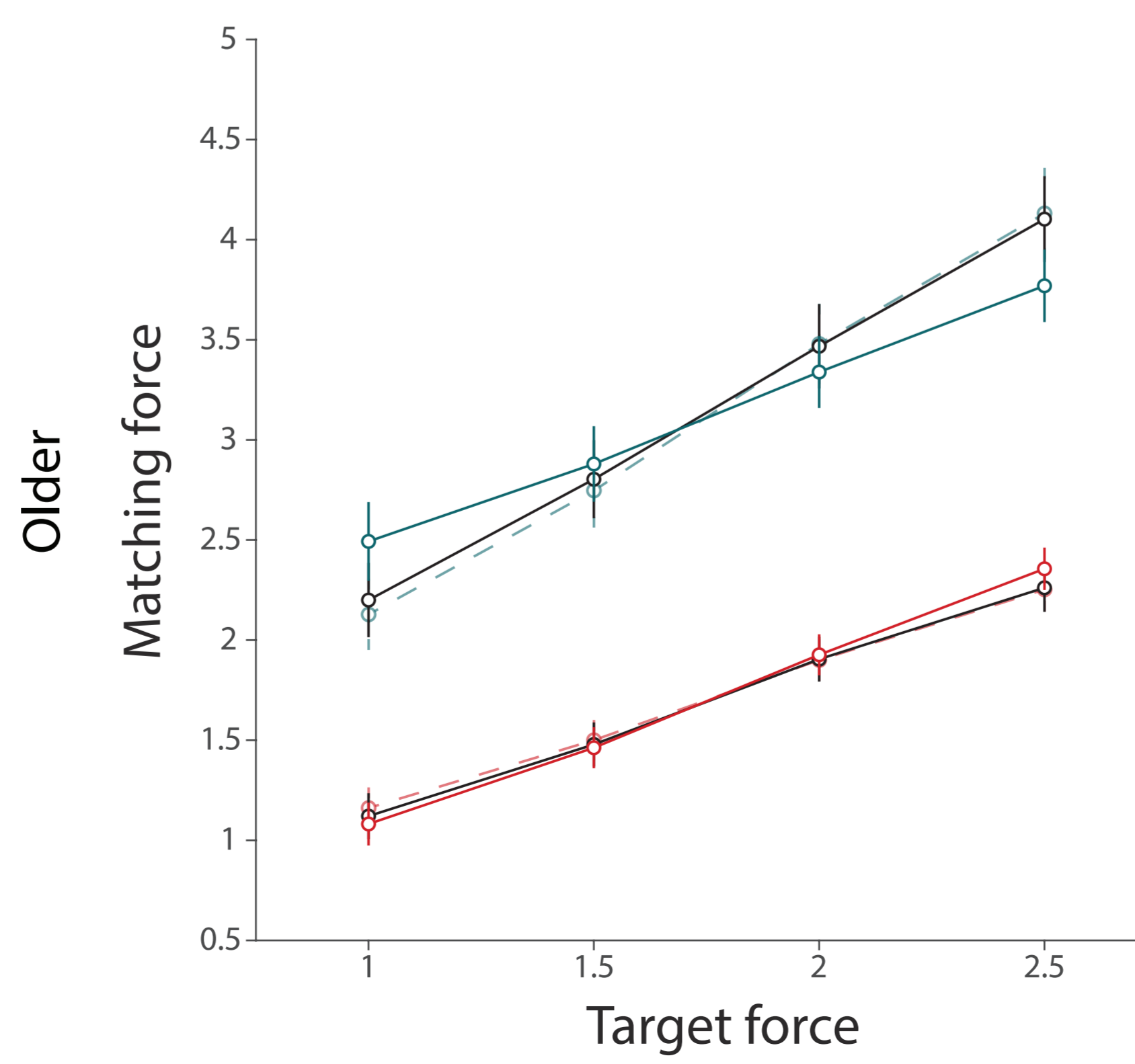

Supplement: Appendix Figure 7 [file EMS206575-supplement-Appendix_Figure_7.pdf]
