# Supplementary material for: Divisive attenuation based on noisy sensorimotor predictions accounts for excess variability in self-touch
Source: J Neurophysiol. Author manuscript; Available in PMC 2025 Jul 28. (PMC7617943; doi:10.1152/jn.00055.2025)
Supplement: Appendix [file EMS206575-supplement-Appendix.docx]

**Appendix**

# **Hierarchical Bayesian modeling**

# Model specification

Figure [A1](#fig:dag) shows a graphical illustration of the hierarchical Bayesian model we fit to empirical force matching data. In brief, for each participant and force level, we fit two parameters (mean and SD) to account for matching forces in the indirect condition. These participant-level parameters were samples from normal and gamma hyper-distributions, respectively, which were unique to each force level but common to all participants. For each participant, we fit two further parameters to account for matching forces in the direct condition across all force levels: these were the mean and SD of the attenuation factor, the key parameters of interest for our analysis. These participant-level parameters were again sampled from normal and gamma hyper-distributions common to all participants. Each parameter associated with a hyper-distribution (nodes outside the participant plate) was constrained by a hyperprior distribution. Below, we provide a full description of the model.

- Figure A1 -

Graph describing the Bayesian hierarchical model. Unshaded and shaded circular nodes indicate unobserved and observed variables, respectively. Single-bordered nodes denote stochastic variables, while double-bordered nodes denote deterministic variables (as the mean direct matching force and standard deviation are fully determined by the stochastic means and standard deviations of the indirect force and attenuation factor). Plates indicate repetitive structures within the model. The additive version of the model is shown: the divisive model has an additional deterministic node corresponding to skewness of direct matching forces.

### Indirect matching forces

We simultaneously fit the data from all conditions within each experiment. Our aim for the indirect reproduction conditions ($I$) was to measure the matching forces rather than to predict them, so we modelled the matching forces on these trials as samples drawn from a normal distribution, with a unique pair of mean and SD parameters for each target force level $t$ and participant:

$$F_{I}^{t}\sim Normal(F_{I}^{t},\sigma_{F_{I}^{t}})$$

Note that for readability we suppress the participant index on all variables. For each participant, we set individual-level priors for the mean and standard deviation within each force level:

$$\begin{aligned} &F_{I}^{t}\sim Normal(\mu_{F_{I}^{t}},1/\sqrt{\tau_{F_{I}^{t}}}) \\ &\sigma_{F_{I}^{t}}\sim Gamma(s_{\sigma_{F_{I}^{t}}},r_{\sigma_{F_{I}^{t}}}) \end{aligned}$$

These individual-level parameters were, in turn, dependent on population-level hyperparameters, with hyperpriors:

$$\begin{aligned} &\mu_{F_{I}^{t}}\sim Normal(F_{T}^{t},1/\sqrt{0.3}) \\ &\tau_{F_{I}^{t}}\sim Gamma(0.001,0.001) \\ \end{aligned}$$

The shape ($s$) and rate ($r$) parameters of the gamma prior distribution on $\sigma_{F_{I}^{t}}$ had priors defined in terms of the central tendency and width of the distribution:

$$\begin{aligned} &\mu_{\sigma_{F_{I}^{t}}}\sim Gamma(0.01,0.01) \\ &\sigma_{\sigma_{F_{I}^{t}}}\sim Gamma(0.001,0.001) \\ \end{aligned}$$

We then used a simple reparametrization to convert the mean and standard deviation into the shape and rate parameters (62):

$$\begin{aligned} &s_{\sigma_{F_{I}^{t}}}=\frac{\mu_{\sigma_{F_{I}^{t}}}^{2}}{\sigma_{\sigma_{F_{I}^{t}}}^{2}} \\ &r_{\sigma_{F_{I}^{t}}}=\frac{\mu_{\sigma_{F_{I}^{t}}}}{\sigma_{\sigma_{F_{I}^{t}}}^{2}} \\ \end{aligned}$$

Parameterizing a gamma distribution in terms of the mean and standard deviation, as done here, results in a much more intuitive interpretation of the prior and resulting posterior distributions than when using shape and rate parameters. However, gamma distributions are typically positively skewed, which can affect their means due to their asymmetric tails. In this case, a potentially better measure of central tendency is the median. Because the median of a gamma distribution has no simple closed form, we opted to define priors over the mean instead. This approach offers a pragmatic compromise, particularly when using weakly informative priors. After fitting the model and obtaining the posterior distributions, we estimated the median numerically by first generating ${10}^{7}$ random samples using the accepted parameters on each MCMC iteration, and then calculating the median of those samples to estimate the posterior distribution of standard deviation of the attenuation. For completeness, we additionally computed the mode using the fitted shape and rate parameters of the gamma distribution (62). Comparisons conducted on the mode estimates were largely consistent with those performed on the median estimates.

### Direct matching forces

**Subtractive model.** In the subtractive model, direct matching forces are considered samples from a normal distribution with mean and SD determined by combining the indirect force matching parameters corresponding to the same target force ($F_{I}^{t}$, $\sigma_{F_{I}^{t}}$) with the attenuation factor parameters ($K$, $\sigma_{K}$), according to Eqs. [1](#eq:addmean)&[2](#eq:addvar):

$$F_{D}^{t}\sim Normal(F_{D}^{t},\sigma_{F_{D}^{t}}).$$

We set individual-level priors for the attenuation parameters:

$$\begin{aligned} &K\sim Normal(\mu_{K},1/\sqrt{\tau_{K}}) \\ &\sigma_{K}\sim Gamma(s_{\sigma_{K}},r_{\sigma_{K}}), \end{aligned}$$

which are again dependent on population-level hyperparameters, with the following hyperpriors:

$$\begin{aligned} &\mu_{K}\sim Normal(1.1,1/\sqrt{0.1}) \\ &\tau_{K}\sim Gamma(0.001,0.001) \\ &\mu_{\sigma_{K}}\sim Gamma(0.01,0.01) \\ &\sigma_{\sigma_{K}}\sim Gamma(0.01,0.01) \\ &s_{\sigma_{K}}=\frac{\mu_{\sigma_{K}}^{2}}{\sigma_{\sigma_{K}}^{2}} \\ &r_{\sigma_{K}}=\frac{\mu_{\sigma_{K}}}{\sigma_{\sigma_{K}}^{2}} \\ \end{aligned}$$

**Divisive model.** In the divisive model, attenuated output force on an individual trial is considered a sample from a skewed-normal distribution (see below) with mean, SD and skewness defined as in Eqs. 3-5:

$$F_{D}^{t}\sim SkewNormal(F_{D}^{t},\sigma_{F_{D}^{t}},Skew[F_{D}^{t}])$$

Individual- and population-level priors for the attenuation parameters and hyperparameters were set identically to the subtractive model (above) except for the population mean hyperprior for $K$ which was set to:

$$\mu_{K}\sim Normal(1.75,1/\sqrt{0.1}).$$

To illustrate the fitted parameters of the divisive model, we plot the mean population-level attenuation factor ($\mu_{K}$) in Figure [5](#fig:postComp)A. Following the described reparametrization of the Gamma distribution, we display the median population-level standard deviation of the attenuation factor ($m_{\sigma_{K}}$) in Figure [5](#fig:postComp)B.

# **Results**

## Linear regression

- Figure A2 -

Matching forces and trial-to-trial variability in matching force in Study 2. (A) Mean matching force as a function of target force, for direct (cyan) and indirect (red) conditions. Data are shown as symbols and linear fits as lines. (B) Matching force in the direct condition plotted against matching force in the indirect condition, with linear fit. Symbols indicate mean matching forces for each target force level. (C) Standard deviation of matching force as a function of target force, for direct (cyan) and indirect (red) conditions, with linear fits. (D) Standard deviation of matching force as a function of mean matching force, with linear fits. Each symbol corresponds to a single target force level. (A-D) Young subjects. (E-H) Same as panels (A-D), but for middle-aged subjects. (I-L) Same as panels (A-D), but for older subjects. Error bars indicate $\pm$1SE, and shaded areas indicate 95% CI.

- Figure A3 -

Matching forces and trial-to-trial variability in matching force in Study 3. (A) Mean matching force as a function of target force, for direct (cyan) and indirect (red) conditions. Data are shown as symbols and linear fits as lines. (B) Matching force in the direct condition plotted against matching force in the indirect condition, with linear fit. Symbols indicate mean matching forces for each target force level. (C) Standard deviation of matching force as a function of target force, for direct (cyan) and indirect (red) conditions, with linear fits. (D) Standard deviation of matching force as a function of mean matching force, with linear fits. Each symbol corresponds to a single target force level. (A-D) Healthy subjects. (E-H) Same as panels (A-D), but for patients subjects. Error bars indicate $\pm$1SE, and shaded areas indicate 95% CI.

## Divisive model

- Figure A4 -

Posterior predictive densities for Study 2. (A) Young subject. (B) Middle-aged subjects. (C) Older subjects. Grey histograms show matching forces pooled across all participants. Coloured distributions show simulated matching forces based on posterior predictive density. The circles with error bars show the median and 90% HDI for data (black) and simulated trials for the same condition (cyan: direct, red: indirect).

- Figure A5 -

Posterior predictive densities for Study 3. (A) Healthy subject. (B) Patients subjects. Grey histograms show matching forces pooled across all participants. Coloured distributions show simulated matching forces based on posterior predictive density. The circles with error bars show the median and 90% HDI for data (black) and simulated trials for the same condition (cyan: direct, red: indirect).

## Subtractive model

- Figure A6 -

Subtractive model posterior predictive densities for Study 1. Grey histograms show matching forces pooled across all participants. Coloured distributions show simulated matching forces based on posterior predictive density. The circles with error bars show the median and 90% HDI for data (black) and simulated trials for the same condition (cyan: direct, red: indirect).

- Figure A7 -

Subtractive model posterior predictive check. (A) Study 1. (B) Study 2 (from top to bottom: young, middle, and older subjects); (C) Study 3 (from top to bottom: healthy and patient subjects). The solid and dashed lines show fits of the subtractive and divisive model, respectively. The circles with error bars show the mean and 95% CI.

Table A1: Summary of Subtractive model

| no | Sample | Attenuation factor | | SD of attenuation factor | | Bayesian *p*-value |
| --- | --- | --- | --- | --- | --- | --- |
|  |  | Median | 90% HDI | Median | 90% HDI |  |
| 1 | Study 1 | 1.36 | [1.24, 1.49] | 0.726 | [0.657, 0.803] | 0.34 *≤*all *p ≤*0.61 |
| 2 | Study 2 young | 0.76 | [0.62, 0.90] | 0.48 | [0.403, 0.569] | 0.43 *≤*all *p ≤*0.54 |
| 3 | Study 2 middle | 1.11 | [1.00, 1.23] | 0.526 | [0.467, 0.586] | 0.40 *≤*all *p ≤*0.57 |
| 4 | Study 2 older | 1.41 | [1.26, 1.55] | 0.646 | [0.567, 0.726] | 0.39 *≤*all *p ≤*0.60 |
| 5 | Study 3 healthy | 1.15 | [0.77, 1.53] | 0.727 | [0.520, 0.955] | 0.39 *≤*all *p ≤*0.55 |
| 6 | Study 3 patients | 0.60 | [0.36, 0.84] | 0.393 | [0.261, 0.528] | 0.46 *≤*all *p ≤*0.52 |

# **Skew-normal approximation to the product of normals**

If$X\sim N(\mu_{x},\sigma_{x})$and$Y\sim N(\mu_{y},\sigma_{y})$, for$Z=XY$,

$$E[Z]=\mu_{x}\mu_{y},$$

$$Var[Z]=\mu_{y}^{2}\sigma_{x}^{2}+\mu_{x}^{2}\sigma_{y}^{2}+\sigma_{x}^{2}\sigma_{y}^{2}=(1+\delta_{x}^{2}+\delta_{y}^{2})\sigma_{x}^{2}\sigma_{y}^{2},$$

$$Skewness[Z]=\frac{6\mu_{x}\mu_{y}\sigma_{x}^{2}\sigma_{y}^{2}}{Var[Z]^{3/2}}=\frac{6\delta_{x}\delta_{y}}{(1+\delta_{x}^{2}+\delta_{y}^{2})^{3/2}}.$$

where$\delta_{x}=\frac{\mu_{x}}{\sigma_{x}}$and$\delta_{y}=\frac{\mu_{y}}{\sigma_{y}}$.

This distribution can be approximated by a skew-normal distribution with the same moments. The skew-normal has pdf,

$$p(x)=\frac{2}{\omega}\phi\left( \frac{x-\xi}{\omega} \right)\Phi\left( \alpha\frac{x-\xi}{\omega} \right).$$

Defining

$$\delta=\frac{\alpha}{\sqrt{1+\alpha^{2}}}\Rightarrow\alpha=\frac{\delta}{\sqrt{1-\delta^{2}}},$$

for$-1<\delta<1$.

$$E[x]=\xi+\omega\delta\sqrt{2/\pi}$$

$$Var[x]=\omega^{2}(1-(2/\pi)\delta^{2})$$

$$Skewness[x]=\gamma_{1}=\frac{4-\pi}{2}(2/\pi)^{3/2}\frac{\delta^{3}}{(1-(2/\pi)\delta^{2})^{3/2}}$$

$$\Rightarrow\delta=Sign(\gamma_{1})\sqrt{\frac{\pi}{2}\frac{|\gamma_{1}|^{2/3}}{|\gamma_{1}|^{2/3}+((4-\pi)/2)^{2/3}}}$$

$$\omega=\sqrt{\frac{Var[x]}{1-(2/\pi)\delta^{2}}}$$

$$\xi=E[x]-\omega\delta\sqrt{2/\pi}.$$
